# Scarcity mindset among schoolteachers: how resource scarcity negatively impacts teachers’ cognition and behaviors

**DOI:** 10.3389/fpsyg.2023.1333735

**Published:** 2024-01-15

**Authors:** Leif Denti, Erik Sturén, Lars-Olof Johansson

**Affiliations:** Department of Psychology, University of Gothenburg, Gothenburg, Sweden

**Keywords:** scarcity mindset, resource scarcity, schoolteachers, work environment, structural equation modeling, cognitive effects

## Abstract

A recent line of research investigates the negative cognitive effects – known as the scarcity mindset – that arise when people face a lack of resources. We expand on this research and show that these cognitive effects are present among Swedish schoolteachers facing a scarcity of time and social resources at work. From an initial interview study we developed novel survey scales to measure teachers’ subjective assessments of available resources and the extent of their scarcity mindset. We then related resource scarcity of time and social resources to the scarcity mindset using structural equation modeling (SEM) analysis in a survey study with a sample of Swedish schoolteachers. This research provides valuable insights for addressing resource constrained work environments in schools and contributes to the broader psychological research on cognitive effects resulting from resource scarcity.

## 1 Introduction

When people believe there is not enough time, space, energy, or money available to complete their tasks efficiently, they tend to focus on what they can accomplish right now rather than planning for the future. According to scarcity theory, a perceived scarcity of vital resources (such as time, social support and material resources) creates a cognitive bias, where attention is narrowed and concentrated on the present (Shah et al., 2012). A constant and prolonged resource scarcity might lead to a negative spiral, a so-called ‘scarcity trap’. Here, people get caught in a cycle of short-term problem solving (commonly known as ‘firefighting’) which in turn lowers their ability to change their situation in the longer term (Mullainathan and Shafir, 2013).

In this paper we present two studies aiming to explain how and why schoolteachers in Sweden are affected psychologically by resource scarcity. In the first study, we interviewed schoolteachers using the Critical Incident Technique (CIT, Flanagan, 1954) to investigate if and how they experienced a scarcity mindset at work, and which type of resources that were salient in their work. In study two, we developed and psychometrically tested four survey scales based on the results of these interviews. Three scales measure the perceived availability of: (1) Time resources, (2) material resources and (3) social-support resources. The fourth scale measured the extent to which participants experienced scarcity-mindset cognitions.

This research contributes to the scientific literature by exploring the under-investigated territory of non-monetary resource scarcity in schools. It extends the scarcity framework, traditionally focused on financial constraints (Shah et al., 2012; Mullainathan and Shafir, 2013; Goldsmith et al., 2020), to the context of Swedish schoolteachers—an important yet often overlooked professional community crucial to societal development. In stress and well-being research, few studies have explored the scarcity mindset within the demanding environments that teachers navigate daily, despite known associations between workplace resources and negative health effects (e.g., Maslach and Leiter, 2016; Bakker and Demerouti, 2017). This study also fills a gap in organizational psychology by developing a novel psychometric instrument that could be used to measure perceived resource scarcity and the associated cognitive mindset in the educational sector. Ultimately, the paper strives to inform policies that can enhance school environments, reduce teacher overload, and improve student outcomes by understanding these dynamics – a call to action supported by the findings of educational effectiveness research (Hattie, 2009).

### 1.1 Scarcity research

This young field of research sheds new light on how our cognition, and by extension, our behavior, is affected when we are placed in situations where we experience resource scarcity. According to scarcity theory, a lack of resources leads to a cognitive state where people direct their focus toward immediately dealing with the scarcity at hand (Shah et al., 2012). This leads to a short-term performance boost (Karau and Kelly, 1992), but also creates a certain set of cognitions labeled the scarcity mindset. This cognitive state can be described as a kind of tunnel vision, in which people direct their attention on their current resource scarcity and ignore activities unrelated to the immediate resource scarcity. In effect, they postpone issues and problems that are less critical in the here and now (Mullainathan and Shafir, 2013). Although previous research have predominantly focused on the effects of monetary resource scarcity (de Bruijn and Antonides, 2021; O’Donnell et al., 2021), showing that a lack of money can lead to sub-optimal decision making, such as taking on loans to pay other loans (Mullainathan and Shafir, 2013), effects of scarcity have also been demonstrated with arbitrary resources such as time, caloric intake and rounds in a computer game (Shah et al., 2012; Mullainathan and Shafir, 2013).

Recent studies have attempted to replicate some of the phenomena suggested by Mullainathan and Shafir in their original conception of scarcity theory (for original studies see mainly Shah et al., 2012; Mani et al., 2013 and Mullainathan and Shafir, 2013; for recent overviews of replications see O’Donnell et al., 2021, and review, see de Bruijn and Antonides, 2021). Replications suggested support for increased attention on scarce resources which can lead to neglect of other information. We have all experienced time scarcity, such as when we need to meet a deadline. Under strong time pressures our attention is directed solely at the task at hand, leading us to neglect other essential information (Shah et al., 2012; de Bruijn and Antonides, 2021). This phenomenon, called attentional neglect, has been demonstrated in various types of resource scarcities (de Bruijn and Antonides, 2021). On a very literal level, attentional neglect has been observed in people failing to see visual information about a discount (Tomm and Zhao, 2016). In this fashion people under scarcity have been shown to engage in a range of counter-productive behaviors like postponing medical appointments, deviating from medication plans, failing to manage their long-term finances and arrive on time for important meetings with authorities (Zwane, 2012; Mani et al., 2013).

Temporal discounting also appears to be an important cognition of the scarcity mindset (O’Donnell et al., 2021). It refers to peoples’ tendency to favor smaller immediate rewards over larger future rewards, a tendency that becomes more pronounced when people are under conditions of scarcity (Bickel et al., 2016). People experiencing scarcity tend to borrow resources from their future to solve their immediate situation (e.g., taking short term high interest blank loans) to the point that they create even more problems for themselves in the future.

The idea that people under resource constraints postpone important problems is central to this study. For example, Perlow (1999) showed how such scarcity thinking can create a negative spiral at the workplace; employees who experienced lack of time considered themselves to be in a crisis situation and focused entirely on solving the most urgent work without paying attention to the planning that could have prevented future crises. In a similar fashion, we expect that teachers who experience resource scarcity will focus on solving immediate problems and ignore long-term efforts and problems. Within the context of Swedish schools, teachers operate in an environment where resource scarcity and health effects is a real concern (Swedish Work Environment Authority, 2014). Time resources, defined not just by the quantity but also the quality of available time, are essential for teachers to prepare lesson plans, provide efficient classroom instruction, and engage in professional development. Swedish teachers have attributed their work situation to unreasonable demands where the work situation is described as a constant struggle for time, having to juggle multiple simultaneous tasks (Månsson and Persson, 2004). In other studies teachers reported high job demands, low social support and high emotional demands (Lindqvist and Nordänger, 2006; Arvidsson et al., 2016). This work situation is one of the primary factors explaining why new teacher graduates choose not to start their careers or resign after a short time in the profession (Struyven and Vanthournout, 2014).

### 1.2 Resources

In this research we draw on Hobfoll (2002) in our definition of resources: resources are means or media that are valued by individuals either for their own sake, or for the opportunity they provide to acquire other things that are valued. Resources can be both objective (i.e., available working time) and subjective (i.e., assessments of the working time available in relation to the job’s perceived difficulty). It is, in other words, not only objective resources, commonly measured as background variables (such as income), that matters. People’s subjective sense of resource scarcity (such as experienced lack of economic resources) may also predict their well-being and future outlook, alongside objective factors. Studies by Einarsdóttir (2018) and Einarsdóttir et al. (2019) found that the most important resource types were socio-emotional, material/economic, and temporal resources. In line with this we investigate in this study how perceptions of three types of resources: social-, time-, and material/economic resources, affect schoolteachers’ scarcity mindset.

Time resources are defined as an individual’s subjective perception of the amount of time available to perform necessary tasks and responsibilities (Hobfoll, 2002). Experiences of time scarcity can therefore arise both from having short deadlines for tasks and from having a large amount of work tasks going on in parallel. For example, Lindqvist and Nordänger (2006) describe how schoolteachers feel compelled to use breaks to deal with extra work that is not related to teaching, such as mediating issues among students and staff. Along similar lines, Johnson (2006) illustrates that teachers at high-poverty schools often face significant time constraints due to their dual roles as educators and quasi-social workers, which leads to a heightened experience of time scarcity.

Social resources are defined as perceived support from supervisors and colleagues, and the perceived quality of relationships with pupils and parents (Hobfoll, 2002). Teachers are exposed to twice as many conflicts and fights as other professional groups and experience less support from management and colleagues (Månsson and Persson, 2004; Swedish Work Environment Authority, 2014). Teachers have also reported that lapses in student discipline are common and require a lot of work, and that the working alliance between teachers, parents and pupils is loosening (Skaalvik and Skaalvik, 2015).

Material resources are defined as the individual’s subjective experience of the availability of monetary or material resources (Hobfoll, 2002). Adequate teaching materials, functioning technology and well-maintained facilities are examples of assets that teachers require to perform their educational roles effectively (Uline and Tschannen-Moran, 2008). When such resources are lacking during lessons, teachers must spend time problem solving, adding to their experience of time scarcity (Johnson, 2006).

## 2 Study 1

Study 1 aimed to collect teachers’ subjective descriptions of their available resources (time, social- and monetary resources), as well as how resource scarcity affected their cognition and behavior. This data was used data to generate survey items for the development of a psychometric survey instrument (Study 2).

### 2.1 Method

#### 2.1.1 Participants

In total we conducted 41 interviews with 24 Swedish primary school teachers and 17 preschool teachers. To be eligible, teachers had to teach at ages 6–15 and work at their school at least part-time. Only public schools and preschools were included. Thirty-two respondents (78%) were women, 9 (22%) were men. This gender percentage roughly matches that of the teacher profession at large in Sweden (SCB, 2022).

#### 2.1.2 Interview technique

Interviews were conducted using the Critical Incident Technique (Flanagan, 1954). We chose this interview technique because the interview data was primarily to be used for generating survey items on scarcity experiences, and CIT being a memory-based interview technique has been reported to increase content validity of descriptions (Butterfield et al., 2005). Participants were prompted to recall a specific situation (incident), whereupon a set of structured interview questions followed. Respondents were instructed to recall three different recent situations in which they experienced (1) a lack of time, (2) lack of social support, and a (3) lack of material resources. Exact wording was *“Can you recall a recent situation in your work where you experienced an acute time crunch/lack of time? It could be not having enough time for your work, for example.”* Participants then answered three follow up questions: (1) *Can you describe the situation?*; (2) *Can you describe how you dealt with it and how you reasoned your way to your decision?* and; (3) *What were the consequences of your behavior?*. Although CIT is based on participants’ recalled memories of specific situations, not all participants could recall such incidents. Rather, they talked more broadly about general types of situations that they meant happened often. For example, some participants talked about a situation in which they felt time scarcity but started mixing in elements from the two other resource types (monetary and social resources) as well. In these interviews, the interviewer made an effort to remain flexible and refrain from intervening.

#### 2.1.3 Procedure

Interviews were conducted at the schools were the respondents worked, commonly in a meeting room or empty classroom. Respondents were given identical instructions verbally, read by the interviewer. They were informed that the aim of the study was to investigate how people reason when trying to handle different kinds of resource scarcities and how they weigh alternatives in their decision making. They were also informed that their personal data were to be confidential. Each interview was audio recorded and transcribed by the interviewer. The transcriptions were done without summarizing or reformulating the interviews.

#### 2.1.4 Analysis

The interview data enabled us to find relevant themes from which survey scale items could be created in Study 2. Since we were interested in covering the constructs broadly, a “basic” thematic method was chosen that is preferable when a large body of data is to be summarized to analyse the interviews (Braun and Clarke, 2006). NVivo (12) was used for all steps of the analysis which in many regards was deductive, i.e., influenced by earlier theory. Interviews were first coded one by one. Relatively “high resolution” coding was used on the semantic (explicit) level, often one sentence at a time, avoiding interpretation. Very little interpretation was done – “reading between the lines” was in other words avoided. The codes were collated into a thematic structure inductively. The final thematic structure was constructed after finishing the interview phase.

### 2.2 Results

## 3 Study 2

In Study 2 we aimed to develop a psychometric instrument, measuring three dimensions of perceived resource scarcity, that is time resources, physical resources and social resources, as well as scarcity cognitions and behaviors in response to these resource scarcities (scarcity mindset).

### 3.1 Method

#### 3.1.1 Item development

Items were generated using the construct map technique (Wilson, 2005), to make sure that the whole continuum of subjective resource scarcity was covered. Construct mapping establishes a wide gamut of the phenomenon being measured to avoid floor and ceiling effects in the final scale. The basic idea is to establish theoretical “end points” of the scale being constructed. This should be done both for persons (subjective situations) and items. This means that when a clear picture of the maximum and minimum amount of the construct emerges, items can more easily be created to fit into this continuum. In our case for example, the maximum amount of scarcity cognitions a person could have would mean an almost complete attention at solving problems created by the scarce resource (theme: tunneling), engaging in short term counter-productive behaviors (theme: borrowing from the future), and cutting corners (theme: resource skimping).

Items were generated using the themes from Study 1 as a point of reference. For each measure, that is, scarcity mindset cognitions, time resources, social resources, and material resources, the process followed a number of divergent (generating) and convergent (pruning) phases. Initially a large number of items were constructed, and each item was further rewritten into three different versions, increasing the richness of item formulations. When this divergent step was done, the pruning phase began. One item was kept from each such triplet resulting in 99 items that formed the basis for a pilot data collection where three teachers gave feedback trough a focus group interview and provided written feedback on items deemed problematic. Based on this pilot study, many items were pruned, and a large number of items were then once again rewritten (Table 1).

**Table 1:** Resource scarcity themes, antecedents and consequences among Swedish schoolteachers.

Theme and definition	Antecedents	Consequences
Time resources		
Lack of time.	(1) Time-consuming unexpected events: pupils fighting in hallways or faulty technical equipment. (2) Time lacking for planning and documentation.	(1) Short term problem solving, less time for long term beneficial tasks such as planning, finding or creating teaching materials, maintaining positive relationships to pupils. (2) Off-hour unpaid work, such as grading or documentation at home.
Physical resources		
Materials Lack of materials.	(1) Lack of teaching materials such as textbooks, exercises, work materials. (2) Lack of equipment such as projectors or lab equipment.	(1) Time spent on copying work materials instead of planning lectures. (2) Time spent outside of work on finding work materials online. (3) Time spent finding equipment.
Personnel resources		
Lack of personnel resources.	(1) Lack of teaching staff and student assistants (2) Lack of school health services	(1) Time spent on managing large classes and disruptive pupils. (2) Time spent on addressing student’s mental or physical issues.
School premises		
Lacking school buildings.	Rundown classrooms and hallways, broken furniture, leaking roofs.	Spending time on finding equipment or rooms, repairing furniture or other equipment.
Social resources		
Management and colleagues		
The degree of supporting and trusting personal relationships with colleagues and management.	Social problems, such as incidents with pupils or parents.	Problems with the principal led teachers to engage in job search behaviors.
Student relationships		
The degree of supporting and trusting personal relationships with pupils.	(1) Classroom size, i.e., number of pupils per classroom. (2) Lack of continuity (teachers moving from class to class). (3) Lack of time for relationship-building.	(1) Disruptive classroom environments. (2) Work spent on solving social problems rather than teaching.
Relationship with parents		
The degree of supporting and trusting personal relationships with parents.	Pressure from parents. Parents in the reported incidents contacted the teachers out of office hours with attempts to influence grades.	(1) Rumination and mental exhaustion. (2) Distancing oneself from work.
Scarcity cognitions		
Tunneling		
A cognitive state where attention is focused on present issues.	(1) Lack of several types of resources: time, materials and social resources. (2) Lack of social resources created the strongest tunneling phenomenon, such as non-trusting relationships with pupils, parents or their principal.	(1) Immediate focus on solving the problem. (2) Less consideration for alternative solutions or strategies. (3) Postponing or ignoring tasks even though teachers knew they were important.
Borrowing from the future		
A behavior in which a future resource (such as time) is used to solve present problems.	Lack of time (e.g., using planning time for more pressing tasks).	(1) Borrowed resources had to be restored at a later point. Often this meant extra work later on in time. (2) Using off-hour time to plan and do administrative tasks.
Resource skimping		
Reducing quality demands to do urgent tasks on time (cutting corners, disobediently ignoring rules).	(1) Lack of time (e.g., less consideration when grading, using old and well-known teaching formats in class). (2) Lack of material resources (e.g., illegally copying teaching materials).	(1) Guilt and discomfort due to doing a worse job than ideal. (2) Stress, rumination and unhappiness when experiencing negative consequences among pupils.

#### 3.1.2 Data collection and preparation

A survey consisting of 99 scarcity mindset items plus control variables was distributed to a sample of 444 teachers in a cross-sectional survey design. To be eligible, teachers had to teach at ages 6–15 and work at their school at least part-time. The control variables were age, gender, years as teacher and approximate class sizes. The Covid-19 pandemic increased the difficulty in recruiting participants, hence data collection was done online, using networks of teachers on Facebook and Linkedin. 121 participants dropped out of the survey at some point; this data was not kept in the final analysis. On the rest of the data, we used the SPSS EM algorithm to impute data that were assumed to be missing at random (>95% survey completion to ensure that we were not imputing entire subscales).

#### 3.1.3 Participants

The final dataset had 323 survey respondents of which 249 were women, 67 men and 3 “other.” Average age was 44.4 (SD = 9.3). On average, the sample had taught for 15.0 years (SD = 8.6). Mean self-reported classroom size was 24.6 pupils (SD = 6.8).

#### 3.1.4 Item pruning through confirmatory factor analysis

Multivariate normality was checked prior to any factor analysis. This was done using the MVN package in R (Korkmaz et al., 2014). Several tests indicated deviations from the expected distributions. The robust “MLR” estimation procedure was therefore used for all factor analyses. Confirmatory factor analysis (CFA) was conducted using lavaan (Rosseel, 2012) in R (version 4.1.2).

Items were in a first step pruned from the scales in a step-wise procedure. Initially, all items were specified to load on a single construct in sub-dimensions that were chosen á priori to reflect the interview themes in Study 1. When pruning items, decisions were guided by the items’ wordings, factor loadings, R-square, modification indices and error variances. If items had low factor loadings and/or modification indices pointed toward cross loadings or large residual correlations the item was removed, and the model was analysed again. If RMSEA and CFI values improved the process was repeated. Latent construct sub-dimensions were also subject to merging, removal and renaming during this process. The rationale for reshaping sub-dimensions were factor loadings, modification indices in combination with an analysis of the semantic meaning of items. The pruning process was repeated until the model consisted of as few items as possible whilst at the same time exhibiting acceptable model fit (CFI > 0.95), as recommended by Hu and Bentler (1999).

### 3.2 Final factor structures and interrelationships

The confirmatory factor analysis resulted in a total of 37 items across three resource scarcity scales and one scarcity cognitions scale. See Supplementary Appendix 1 for item wordings and factor loadings. The final solutions for all scales had decent model fit. Robust chi-square, CFI and TLI can be seen in Table 2. RMSEA varied between an acceptable 0.034 for the social resources scale and a relatively high 0.109 for the time resources scale.

**Table 2:** Model-fit metrics for the four latent constructs.

	Robust chi-square (*df*)	CFI	TLI	RMSEA
Scarcity mindset	85.177 (*27*)	0.945	0.924	0.092
Physical resources	28.736 (*17*)	0.975	0.960	0.048
Social resources	70.051 (*55*)	0.990	0.986	0.034
Time resources	63.409 (*14*)	0.947	0.921	0.109

**Figure 1:**
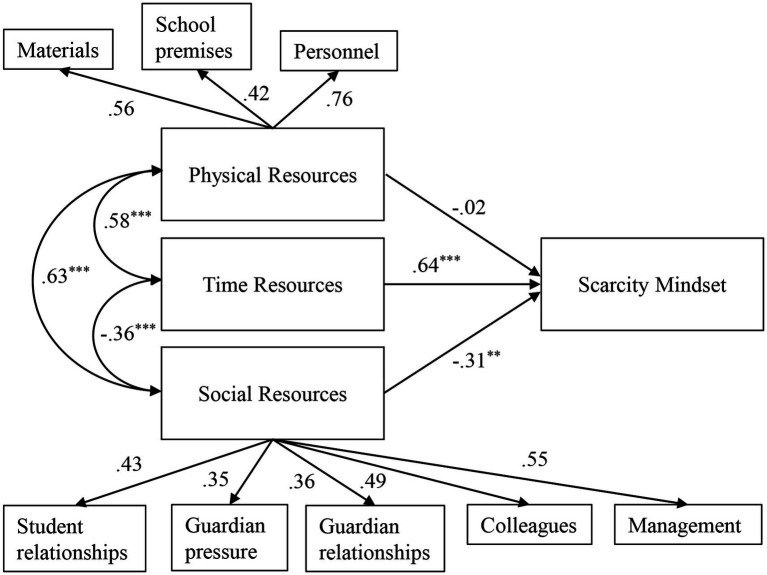
Final structural equation model. Physical resources, social resources and time resources predicted scarcity mindset. All predictors were allowed to covary. Significance for regressions indicated at 0.05, 0.01, and 0.001 levels with ^*^, ^**^, and ^***^, respectively.

Next, we specified a structural equation model to investigate if the resource scarcity scales predicted variance in the scarcity cognitions scale (criterion). Latent variables for the three resource scales were regressed directly onto a latent variable for scarcity cognitions. The full model with standardized factor loadings, regressions and covariances can be seen in Figure 1. This model had reasonably good fit, *χ*^2^ (615) = 1023.34 (*p* < 0.001), *CFI* = 0.913, *TLI* = 0.906, *RMSEA* = 0.047. Items overall had good factor loadings (*unstandardized factor loading mean* = 0.979) and *R*^2^- values (*mean* = 0.493). The lowest *R*^2^-values were among some of the items in the physical and social resources scales. There were six instances where the SEM model reported modification indices >15, with the largest being 23.334. Three of these concerned the social resource scale. The mean standardized residual for all scales as a whole was still a reasonable 0.646.

Regressions indicated that the social and time latent factors influenced the amount of scarcity mindset, where a lower level of perceived resources was related to more scarcity experiences in both cases. Both of these regressions were significant (social resources: standardized *β* = −0.324, *p* = 0.023, time resources: standardized *β* = 0.632, *p* < 0.001). The directions were as expected considering the item wordings. The regression for the physical scale was not significant (standardized *β* = −0.012, *p* = 0.930). There was however a standardized covariance of −0.572 between the time and physical scales (Table 3).

**Table 3:** Means, standard deviations, skewness, kurtosis and correlations among study variables.

Scale	*M*	*SD*	Skew	Kurtosis	1	2	3
1. Scarcity mindset	2.54	0.87	0.48	−0.11			
2. Physical resources	3.30	0.71	−0.23	−0.34	−0.45^**^		
3. Social resources	1.97	0.55	0.70	1.03	−0.42^**^	0.38^**^	
4. Time resources	1.97	0.81	1.2	1.62	0.65^**^	−0.47^**^	−0.28^**^

*M* and *SD* are used to represent mean and standard deviation, respectively. Significance indicated at 0.05 and 0.01 levels with ^*^ and ^**^, respectively.

## 4 Discussion

This study aimed to identify teachers’ subjective experiences of resource scarcity and explore if their scarcity perceptions can be linked to a “scarcity mindset” – a cognitive state that entails three themes: attentional tunnel vision, borrowing from the future, and resource skimping.

Study 1 showed that teachers described themselves being ensnared in a perpetual cycle of immediate problem-solving, commonly known as “firefighting,” to the detriment of long-term planning and quality teaching. They report large deficits in available resources in their environments, often keeping them from carrying out their teaching activities. Teachers in this study also reported that they constantly had to cut corners, lower output quality and practice letting things go. Concretely, educators reported instances of being unable to adequately prepare for lessons due to emergent discipline issues or administrative tasks, consistent with Perlow’s (1999) findings on workplace crisis mentality. The dedication to immediate needs remains a salient theme consistent with the scarcity literature’s emphasis on tunnel vision and neglect of broader considerations (Shah et al., 2012). Furthermore, teachers shared stories of emotional fatigue stemming from a lack of social-support resources, particularly when they lacked immediate access to support staff or colleagues, a condition aligning with Månsson and Persson’s (2004) work on the importance of managerial and collegial support in schools. In summary, our interviews consistently showed that working conditions were not sustainable; many participants had been burned out once or were certain they were at high risk of burnout and almost all participants reported working unpaid overtime.

Study 2 corroborates these results in that teachers reported high workload and high scarcity of all relevant resources measured in the survey. The results from our SEM model supports our initial reasoning, that a lack of various types of resources is related to a higher amount of self-reported attentional tunnel vision, temporal discounting and corner cutting at work. These findings are consistent with phenomena described in the scarcity literature, for example Tomm and Zhao (2016) and Shah et al. (2012).

A novel contribution from this study is the demonstration that resource scarcity and the scarcity mindset can be operationalized and measured in a school context using survey items. Our SEM model supports the idea that the resources teachers manage at work are related, but function independent of each other. Factor structures of the survey items showed good fit to the data. In support of instrument validity, between-factor relations were statistically significant, indicating that the expected effects shown in previous experimental studies were present here as well. Measuring the scarcity mindset using our survey scale is therefore a viable route for future studies on scarcity.

Since previous studies have focused mainly on scarcity of money and time as antecedents to the scarcity mindset, we contribute to theory by showing that scarcity cognitions can emerge from a lack of social resources in schools. Social resource scarcity exhibits a significant negative relationship with scarcity cognitions in our study. This extends the work of Hobfoll (2002) on the significance of social support as a crucial resource predicting scarcity cognitions and underscores the role of interpersonal relations (Månsson and Persson, 2004) within the literature of educator job satisfaction and stress (Skaalvik and Skaalvik, 2015).

### 4.1 Limitations

First, the recruitment of participants through social media (due to Covid-19) resulted in a somewhat smaller sample size than planned. This may to some extent limit our interpretations since simulation studies have suggested that a model of the size tested ideally should have a larger sample (Wolf et al., 2013). However, given the lack of previous research specifying what to expect in terms of effect sizes and explained variance, it is impossible to know the required sample size beforehand. Yet, our model test suggest that the sample size was large enough to produce robust results (see Figure 1). Nevertheless, further studies should aim to test our findings using a larger sample. Second, although schools were generally open in Sweden during the Covid-19 pandemic, some schools with pupils aged 13–15 were partially closed. This might have caused the non-significant relationship between physical resources and scarcity mindset. A third limitation is that the item wordings in our survey scale may overlap with other constructs, such as various types of stress. A high workload, or lack of time to do work tasks as formulated in the time resources scale, will likely be related to psychological stress. As of now we do not have data to test the potential overlaps between this scale and related constructs. This needs to be addressed in future research.

### 4.2 Future research

Our findings support that, teachers’ perceptions of time and social resources are clearly associated with their levels of scarcity mindset. A direction for future research is to further examine which antecedent resource types are associated to scarcity cognitions and behavior, and if there are mediators in this causal chain. For example, time scarcity may be considered a late-stage mediator affecting scarcity cognitions and behavior, with social and material resources as distal predictors. Results from our interview study indicated for example that physical resource scarcity might affect time resources negatively (a lack of books or equipment may for example force teachers to copy lecture material and/or move to lecture rooms with the appropriate equipment), which in turn may give rise to scarcity cognitions and behaviors. Another example is staffing, where a lack of teacher resources or student assistants force teachers to focus on disruptive pupils in large classes.

Another venue for future research is to study the consequences of the scarcity mindset at the individual, team and organizational levels. These consequences might be behavioral, cognitive as well as affective. At the individual level, such consequences can be procrastination (behavior), short sightedness (cognition), as well as increased stress and decreased subjective well-being (affective) (Hunter and Wu, 2016). At the team level, previous research has found that cooperation behaviors decrease under scarcity, possibly affecting collegiality and citizenship negatively (Perlow, 1999; Prediger et al., 2014; Roux et al., 2015). At the organization level, diminished change readiness, absorptive capacity and innovation may be consequences of high scarcity cognitions and behaviors across employees, since those outcomes typically demand long-term planning (Lavie et al., 2010).

### 4.3 Implications for practitioners

These findings have implications for educational policy and school administration. The interview material in Study 1 showed that different resource types interacted, sometimes in complex patterns. For example, low quality relationships with pupils (social resources) meant that teachers spent time creating order in classrooms and hallways – time taken from lecturing. Thus, efforts to increase overall school quality by alleviating resource problems need to have a holistic perspective, considering several resource types and how they interact.

Increasing the amount of time available is likely crucial to any real change in teachers’ experiences of school quality. More slack time, that is the extra time that may be available to complete a task – in this case having time to deal with unexpected situations in classrooms or in corridors – might help to temper the scarcity mindset. In addressing time scarcity we suggest school leaders should consider areas such as workload management and scheduling. The problems related to a scarcity mindset can be reduced by distributing time-consuming activities throughout the year, thereby easing conflicts between short- and long-term tasks. Given that teachers under scarcity conditions may borrow time from or altogether neglect activities with long-term returns, planning and curriculum development should ideally not occur concurrently with short-term time-consuming activities such grading national tests or exams. Moreover, we suggest that time-saving activities such as AI-tools, administrative support or relief from administrative duties could directly impact teachers’ time budgets. Learning effective teaching methods could also free up valuable time resources in the classroom (Hattie, 2012).

Mitigating social resource scarcity entails fostering a more collaborative culture. Distributed leadership and shared decision-making structures may both foster collaboration and reduce teacher workload (Harris and Jones, 2020). Learning effective crisis and conflict management skills might also help both school leaders and staff navigate social incidents more successfully. Peer mentoring programs and professional learning communities may also increase collegial support and provide platforms for problem-solving and learning (Voelkel Jr. and Chrispeels, 2017). Strengthening the teacher-parent relationship is also critical. As Bryk and Schneider (2002) showed in a three year study of 12 elementary schools, fostering trust and cooperation between schools and parents through structured communication and community-building practices might alleviate teachers’ stress and contribute to positive learning environments. Methods for student participation might also contribute to shared understanding and cooperation between teachers and students (Pascual-Arias et al., 2022).

## 5 Conclusion

This study reveals that Swedish teachers’ experience of scarcity in time and social resources contributes to a scarcity mindset. Teachers reported that these shortages lead to less effective teaching, lower quality relationships with pupils and an overall inefficient use of time. Addressing these scarcities through targeted policies and organizational change has the potential to enhance teachers work situation and school quality. Our results also demonstrate how different types of resources are interconnected and may influence each other. For example, we observe that providing teachers opportunities to improve relationships with pupils might also help address time constraints for other tasks. It is important that school policy and organization are holistic, recognizing the interconnectedness of resources to optimize the school environment effectively.

## Data availability statement

The raw data supporting the conclusions of this article will be made available by the authors, without undue reservation.

## Ethics statement

The studies involving humans were approved by Swedish Ethical Review Authority. The studies were conducted in accordance with the local legislation and institutional requirements. The participants provided their written informed consent to participate in this study.

## Author contributions

LD: Writing – original draft, Writing – review & editing. ES: Writing – original draft. L-OJ: Writing – original draft, Writing – review & editing.
