# Supplementary material for: Scarcity mindset among schoolteachers: how resource scarcity negatively impacts teachers’ cognition and behaviors
Source: Front Psychol. 2024 Jan 15;14:1333735. doi: 10.3389/fpsyg.2023.1333735 (PMC10822981; doi:10.3389/fpsyg.2023.1333735)
Supplement: Supplementary file 1 [file Table_1.docx]

Supplementary Appendix 1

*Item wordings, subscales and standardized loadings.*

| Scarcity mindset cognitions and behaviors | | Loading |  |  |  |
| --- | --- | --- | --- | --- | --- |
| 1 | I often have to focus on one thing at a time to get through the day. | 0.672 |  |  |  |
| 2 | I find it difficult to get an overall picture of all my tasks. | 0.661 |  |  |  |
| 3 | I am often forced to do a worse job than I would really like. | 0.763 |  |  |  |
| 4 | My focus only extends only one hour ahead in time. | 0.650 |  |  |  |
| 5 | I often find it difficult to shift my focus from one task to another. | 0.734 |  |  |  |
| 6 | I usually focus only on what needs to be done at the moment. | 0.663 |  |  |  |
| 7 | I put off things and problems that need to be solved in the long term. | 0.706 |  |  |  |
| 8 | Short-term, urgent tasks crowd out what I need to do in the longer term. | 0.687 |  |  |  |
| 9 | I can't focus on one thing at a time and have fragmented attention. | 0.702 |  |  |  |
|  | |  |  |  |  |
| Physical resources | | Loading | Subscale |  |  |
| 1 | I have access to all the learning materials and extras I need for my teaching. | 0.782 | Materials |  |  |
| 5 | I have to spend a lot of time copying or otherwise creating learning materials (copying or searching on the internet etc.). | 0.581 | Materials |  |  |
| 8 | All pupils have access to their own textbook. | 0.533 | Materials |  |  |
| 2 | The school health service can help whenever I feel that a pupil needs support. | 0.645 | Personnel |  |  |
| 3 | We have good access to teachers or other teaching staff in the school. | 0.676 | Personnel |  |  |
| 4 | All pupils who need a student assistant have one. | 0.632 | Personnel |  |  |
| 6 | The property manager takes care of the maintenance of the building in time so that problems do not arise. | 0.766 | School premises |  |  |
| 7 | The standard of the premises is very high. | 0.594 | School premises |  |  |
|  | |  |  |  |  |
| Social resources | | Loading | Subscale |  |  |
| 1 | I can trust my colleagues. | 0.756 | Colleagues | |  |
| 2 | I get help from my colleagues when I need it. | 0.836 | Colleagues | |  |
| 3 | I have a good working relationship with my pupils. | 0.784 | Student relationships | |  |
| 4 | I have developed a positive and supportive relationship with my pupils. | 0.700 | Student relationships | |  |
| 5 | Pupils listen to me and do what I say. | 0.581 | Student relationships | |  |
| 6 | Guardians try to influence how I do assessments or grading (R). | 0.688 | Guardian pressure | |  |
| 7 | I have a working relationship with my pupils' carers. | 0.792 | Guardian relationships | |  |
| 8 | I have effective communication with pupils' carers. | 0.902 | Guardian relationships | |  |
| 9 | There are threats from pupils' guardians (R). | 0.637 | Guardian pressure | |  |
| 10 | I feel that guardians do not respect my professional competence (R). | 0.732 | Guardian pressure | |  |
| 12 | I have good communication with my principal. | 0.906 | Management | |  |
| 13 | Because of my principal, I would consider changing jobs (R). | 0.881 | Management | |  |
| 14 | I trust my principal. | 0.920 | Management | |  |
|  | |  |  |  |  |
| Time resources | | Loading |  |  |  |
| 1 | I don't have time to do everything I need to do in the time I have available. | 0.593 |  | |  |
| 2 | I am constantly short of time. | 0.902 |  | |  |
| 3 | I would like to do more but I don't have the time. | 0.820 |  | |  |
| 4 | I have to work extra outside paid working hours to do all my tasks. | 0.717 |  | |  |
| 5 | I often need to spend time outside my working day dealing with situations that arise | 0.558 |  | |  |
| 6 | I rarely have time to plan and revise lessons in my planning time. | 0.747 |  | |  |
| 7 | I have time in my schedule that I can use to plan or solve unforeseen problems. | 0.468 |  | |  |

*Note.* Reverse coded items are denoted with (R).
